# Hypoxia mediated isolation and expansion enhances the chondrogenic capacity of bone marrow mesenchymal stromal cells

**DOI:** 10.1186/scrt100

**Published:** 2012-03-02

**Authors:** Adetola B Adesida, Aillette Mulet-Sierra, Nadr M Jomha

**Affiliations:** 1 Department of Surgery, Division of Orthopaedic Surgery, University of Alberta, Edmonton AB T6G 2R3, Canada

**Keywords:** Chondrogenesis, chondrocytes, hypoxia, bone marrow stem cells, tissue engineering, cartilage repair

## Abstract

**Introduction:**

The capacity of bone marrow mesenchymal stromal cells (BMSCs) to be induced into chondrocytes has drawn much attention for cell-based cartilage repair. BMSCs represent a small proportion of cells of the bone marrow stromal compartment and, thus, culture expansion is a necessity for therapeutic use. However, there is no consensus on how BMSCs should be isolated nor expanded to maximize their chondrogenic potential. During embryonic development pluripotent stem cells differentiate into chondrocytes and form cartilage in a hypoxic microenvironment.

**Methods:**

Freshly harvested human BMSCs were isolated and expanded from the aspirates of six donors, under either hypoxic conditions (3% O_2_) or normoxic conditions (21% O_2_). A colony-forming unit fibroblastic (Cfu-f) assay was used to determine the number of cell colonies developed from each donor. BMSCs at passage 2 (P2) were characterized by flow cytometry for the phenotypic expression of cell surface markers on mesenchymal stem cells. BMSCs at P2 were subsequently cultured *in vitro *as three-dimensional cell pellets in a defined serum-free chondrogenic medium under normoxic and hypoxic conditions. Chondrogenic differentiation of the BMSCs was characterized by biochemical and histological methods and by quantitative gene-expression analysis.

**Results:**

After 14 days of culture, the number of BMSC colonies developed under hypoxia was generally higher (8% to 38% depending on donor) than under normoxia. BMSCs were positive for the cell surface markers CD13, CD29, CD44, CD73, CD90, CD105 and CD151, and negative for CD34. Regardless of the oxygen tension during pellet culture, hypoxia-expanded BMSC pellets underwent a more robust chondrogenesis than normoxia-expanded BMSC pellets after three weeks of culture, as judged by increased glycosaminoglycan synthesis and Safranin O staining, along with increased mRNA expression of aggrecan, collagen II and Sox9. Hypoxic conditions enhanced the mRNA expression of hypoxia inducible factor-2 alpha (HIF-2α) but suppressed the mRNA expression of collagen X in BMSC pellet cultures regardless of the oxygen tension during BMSC isolation and propagation.

**Conclusions:**

Taken together, our data demonstrate that isolation and expansion of BMSCs under hypoxic conditions augments the chondrogenic potential of BMSCs. This suggests that hypoxia-mediated isolation and expansion of BMSCs may improve clinical applications of BMSCs for cartilage repair.

## Introduction

Articular cartilage covers the end of long bones in articulating joints where it provides near frictionless movement. Unfortunately, articular cartilage has a very limited capacity to repair after injury. If left untreated, cartilage defects progressively lead to more extensive lesions and, ultimately, require joint arthroplasty. Cell-based strategies using culture expanded autologous chondrocytes from non-loading regions of articular cartilage are currently used to treat focal cartilage defects [1]. However, there is some evidence of progressive degenerative changes in the joint using this technique [2]. Furthermore, there is evidence that the matrix-forming capacity of expanded chondrocytes is compromised due to de-differentiation processes [3,4]. Thus, there is interest in other cell sources for cartilage repair.

Adherent bone marrow stromal cells or bone marrow mesenchymal stromal cells (BMSCs) have received much interest for cartilage repair because of their multipotent capacity to differentiate into different cell types including chondrocytes [5-10]. While there has been much study related to the potential of BMSCs to form cartilaginous tissue, there has been a limited number of reports of the implantation of human BMSCs for cartilage repair [11]. The reason for this is unclear but may be related to hypertrophic differentiation or the lack of a consensus on how human BMSCs are to be cultured for reproducible and optimal chondrogenic differentiation. Human BMSCs have been estimated to account for a mere 0.001% to 0.01% of the total bone marrow mononuclear cells (MNCs) in the stromal compartment of bone marrow [5,12,13]. Thus, *in vitro *culture expansion is a requisite for increasing cell numbers for research and clinical applications. Since the first published report of Friedenstein and co-workers [14], describing the isolation and expansion of an adherent and spindle-shaped population of cells from whole human bone marrow aspirates, little has changed in the methodology of isolation and expansion of BMSCs. While it is practiced within the art that human BMSCs are isolated after initial cell adherence to tissue culture plastic ware and subsequent cell expansion under normal mammalian conditions of air containing 21% oxygen tension (normoxia), there is increasing evidence that BMSCs are adapted to limiting metabolic conditions [15]. In agreement with this observation, hypoxic (3% O_2_) conditions have been reported to favor the multi-potentiality of a subpopulation of human bone marrow stromal cells over osteogenic differentiation [16]. Accordingly, human BMSCs showed enhanced proliferative activity under hypoxic (1.5% to 3% O_2_) conditions relative to normoxia [16-18]. While these studies demonstrated the effect of hypoxia on human BMSC expansion *in vitro*, the downstream effect of hypoxic conditions during isolation and expansion on human BMSCs' chondrogenic differentiation capacity was unexplored. D'Ippolito *et al. *[16] and Grayson *et al. *[18] investigated osteogenic differentiation of BMSCs after hypoxia mediated expansion, while the study of Martin-Rendon *et al. *[17] investigated chondrogenesis on commercially acquired human BMSCs that lacked initial isolation and cell expansion culture history prior to further cell propagation under normoxia and subsequent chondrogenic differentiation under normoxia and hypoxia for their studies; hypoxia enhanced BMSC chondrogenic differentiation potential.

Ovine BMSCs have been isolated and propagated under hypoxia (5% O_2_) and shown to increase in proliferation rate relative to cells expanded under normoxia. Moreover, it was demonstrated that ovine BMSCs isolated and expanded under hypoxic conditions and subsequent chondrogenic differentiation under normoxia displayed an enhanced chondrogenic phenotype compared to their counterparts after normoxia mediated isolation and expansion [19]. However, it is not clear whether hypoxia isolated and propagated ovine BMSCs would have similarly displayed improved chondrogenesis had they been differentiated under hypoxic conditions.

While it has been shown that normoxia isolated and expanded non-human and human BMSCs undergo enhanced chondrogenic differentiation under hypoxic conditions [9,17,20], it is unknown whether human BMSCs isolated and expanded under hypoxia are predisposed towards improved subsequent chondrogenesis regardless of the oxygen tension. The study of Mueller *et al. *[20], showed that hypoxic conditions during expansion culture of human BMSCs resulted in improved chondrogenesis even under normoxic conditions. However, the study used BMSCs that had been initially isolated and expanded under normoxia prior to further culture expansion under hypoxia and subsequent chondrogenic differentiation culture under hypoxia and normoxia. Perhaps the improved chondrogenesis noted by Mueller *et al. *was not surprising because, during embryonic development, cartilage formation occurs when mesenchymal stem cells condense and differentiate into chondrocytes during an avascular period in a low oxygen tension microenvironment [21]. Thus, oxygen tension is known to play a significant role in the fate of mesenchymal stem cells.

In this study, we investigated the effect of oxygen tension from the onset of human BMSCs isolation and expansion on subsequent *in vitro *chondrogenesis under normoxic and hypoxic conditions. We hypothesized that persistent hypoxic culture during human BMSC isolation and expansion enhances the chondrogenic differentiation capacity under normoxia and hypoxia by altering the expression of genes that facilitate chondrogenesis.

Typically, *in vitro *chondrogenesis of BMSCs is performed in 3D cell pellet culture, in order to mimic mesenchymal cell condensation during chondrogenesis in embryonic development, in the presence of serum free chondrogenic factors comprising TGF-β1 or -β3, dexamethasone, ascorbate and ITS+1 under normoxic conditions [6,9,10,19,22-25]. In this study, we adopted the pellet culture method to investigate *in vitro *chondrogenesis of both normoxia and hypoxia isolated and expanded human BMSCs. *In vitro *chondrogenesis was implemented under normoxic conditions only, in order to isolate the downstream effect of cell isolation and expansion under hypoxic conditions on BMSC chondrogenesis; several studies have reported that hypoxic culture conditions enhance chondrogenic differentiation [9,20,26-32].

## Materials and methods

### Collection of bone marrow specimens and culture bone marrow stem cells

**Figure 1:**
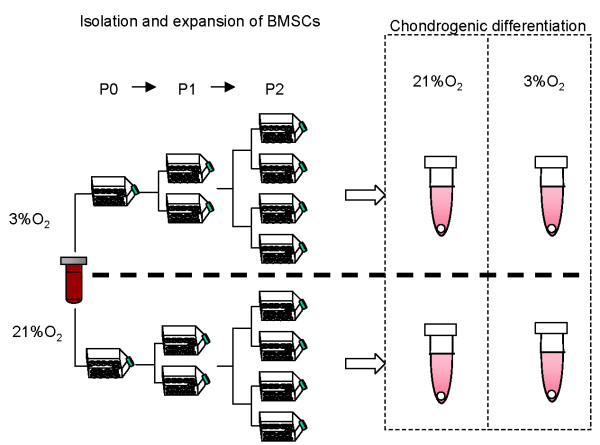
**Experimental set up for isolation, expansion and *in vitro *chondrogenesis of bone marrow mesenchymal stromal cells (BMSCs)**.

Bone marrow aspirates were obtained from surgically discarded material after approval and a waiver of informed consent of the local ethical committee of the University of Alberta (Edmonton, Canada) during orthopedic procedures from the iliac crest of six donors (Table 1; one woman, 34 years old, and five men, 43 to 62 years old). The number of nucleated cells in the aspirates was determined by crystal violet nuclei staining and cell counting using a hemacytometer. Thereafter, 15 million mono-nucleated cells (MNCs) were seeded per 150 cm^2 ^tissue culture flask. The culture medium was αMEM supplemented with 10% heat inactivated fetal bovine serum, penicillin-streptomycin, 4-(2-hydroxyethyl)-1-piperazineethanesulfonic acid (HEPES), sodium pyruvate (all from Invitrogen, Mississauga, Ontario, Canada) and 5 ng/ml basic fibroblast growth factor (bFGF or FGF2; from Humanzyme, Medicorp Inc., Montreal, Quebec, Canada) in order to maintain pluri-potency [33]. Nucleated cells were allowed to adhere and grow for seven days before the first media change under normoxia (ambient oxygen tension of 21%) or hypoxia (low oxygen tension of 3% O_2_) at 37°C in a humidified incubator with 5% CO_2_. Thereafter, the media were changed twice per week until 70% to 80% cell confluence was obtained. However, during feeds hypoxia cultivated cells experienced short periods (< 5 minutes) of re-oxygenation. These adherent bone marrow stromal cells (BMSCs) were detached using 0.05% trypsin-ethylenediaminetetraacetic acid (EDTA) (Sigma-Aldrich, Oakville, Canada) and expanded under normoxia or hypoxia until passage 2 prior to experimental use. The time taken from plating of nucleated cells (P0) to reach approximately 80% confluence at passage 2, before experimental use, varied three to four weeks and was donor and oxygen tension dependent. Both normoxia- and hypoxia-expanded BMSCs from the same donor were kept in culture for the same duration until experiments. The experimental set up for BMSC isolation, expansion and subsequent chondrogenic differentiation is illustrated in Figure 1.

**Table 1:** Donor information of bone marrow aspirates from the iliac crest

Donor	Age	Gender
BM68	34	Female

BM69	46	Male

BM73	51	Male

BM74	43	Male

BM79	45	Male

BM84	62	Male

### Colony forming unit fibroblastic (cfu-f) assay

The proportion of adherent cell population within each bone marrow aspirate specimen was assessed by Cfu-f assay. Cfu-f was performed by plating 2.5 × 10^5 ^nucleated cells in 100 mm sterile petri dishes in triplicate (Becton Dickinson, Mississauga, Ontario, Canada). The cells were cultured as described before using αMEM supplemented with 10% heat inactivated fetal bovine serum, penicillin-streptomycin, HEPES, sodium pyruvate and 5 ng/ml FGF-2 for two weeks under normoxia or hypoxia. After the first week, the non-adherent cell population was removed by aspiration and culture media were replenished every three days until two weeks of culture. During feeds, hypoxia cultivated cells experienced a short period (< 5 minutes) of exposure to normal oxygen tension. After two weeks the developed cell colonies were visualized after fixing with 4% phosphate buffered formalin, washing by PBS and staining using 0.25% crystal violet solution (Sigma-Aldrich). The number of cell colonies developed was recorded as well as their respective diameters. Data were compared in a Student *t*-test and a significant difference between the two culture conditions was considered when *P *< 0.05.

### Flow cytometry analysis

All primary monoclonal antibodies used herein were directly conjugated antibodies to fluorescein isothiocyanate (mAb-FITC) or to phycoerythrin (mAb-PE). Antibodies were either from BD Pharmingen or Invitrogen-see Table 2. The cells were analyzed on a FACScan flow cytometer (Becton Dickinson) after detachment from the culture flask by 0.05% w/v trypsin-EDTA (Sigma). Staining buffer was prepared with PBS containing 1% w/v BSA (Sigma). Normoxia and hypoxia expanded BMSCs were re-suspended in 4°C cold staining buffer at 5 × 10^6^/ml. The cells were dispensed into sample tubes (12 × 75 mm polystyrene round-bottom tubes, Becton Dickinson) in 20 μl aliquots and incubated for 15 minutes with the antibodies at 4°C. All incubations were implemented in 5 ml dilution tubes at room temperature in the dark and all washing steps were performed by a combination of centrifugation (400 g, 5 minutes) and aspiration of supernatant. Staining buffer (200 μl) was then added to the tubes and the cells were incubated for 10 additional minutes. After removal of the supernatant by centrifugation, cells were washed with PBS and kept cold before analysis by flow cytometry. Non-specific staining was assessed using relevant isotype controls. Single color immunofluorescence analysis for the different surface markers was performed with mAb-FITC and mAb-PE. Data acquisition was performed with Cellquest software (Becton Dickinson). FITC emission was measured at FL1 and PE at FL3. For each sample a region for live cells was defined, according to the Forward Scatter (FSC) and Side Scatter (SSC) signals, which excluded aggregated cells from the analysis. Data analysis was performed with Cyflogic version 1.2.1, Perthu Terho &^©^CyFlo Ltd., Finland. For each surface marker analyzed, the percentage of positive cells and the level of marker expression were calculated. The percentage of positive cells was calculated as the percentage of cells having a measured fluorescence greater than that of 99.5% of the cells stained with each associated isotype control. Cells were considered positive for a surface marker when the percentage of positive cells for that surface marker was ≥ 6%. The level of expression of each marker was calculated as the ratio between geometric mean fluorescence intensity (MFI) of samples and that of the isotype control.

**Table 2:** Antibodies used to characterize normoxia and hypoxia expanded BMSC

Specificity	Isotype^a^	Cat.#/Flurochrome	Source
CD13 (aminopeptidase-n)	mIgG1	sc-70529/PE	Santa Cruz Biotechnology
CD29	mIgG1	CD2901/FITC	Invitrogen
CD34	mIgG1	sc-19621/FITC	Santa Cruz Biotechnology
CD44 (Pgp-1, H-CAM, Ly 24)	mIgG2b	560977/FITC	BD Pharmingen
CD73	mIgG1	550257/PE	BD Pharmingen
CD90 (Thy-1)	mIgG1	55596/PE	BD Pharmingen
CD 105 (endoglin)	mIgG1	sc-71043/PE	Santa Cruz Biotechnology
CD151 (PETA-3)	mIgG1	556057/PE	BD Pharmingen
Not specified (Isotype control)	mIgG1	sc-2855/FITC	Santa Cruz Biotechnology
Not specified (Isotype control)	mIgG1	sc-2866/PE	Santa Cruz Biotechnology
Not specified (Isotype control)	mIgG2b	sc-2857/FITC	Santa Cruz Biotechnology
Mouse pooled immunoglobulin (Isotype control)	rIgG2a	554688/FITC	BD Pharmingen
TNP-KHL (Isotype control)	mIgG2a	555575/PE-Cy™5	BD Pharmingen

^a^m, mouse; r, rat. BMSC, bone marrow mesenchymal stromal cells; CAT.#, catalogue number.

### *In vitro *chondrogenic differentiation

BMSCs at passage 2 (P2) were re-suspended in chondrogenic culture medium consisting of high glucose (D)MEM containing 4.5 mg/ml D-Glucose, 0.1 mM non-essential amino acids, 1 mM sodium pyruvate, 100 mM HEPES buffer, 1 mM sodium pyruvate, 100 U/ml penicillin, 100 μg/ml streptomycin, 0.29 mg/ml L-glutamine (all from Invitrogen) supplemented with 0.1 mM ascorbic acid 2-phosphate, 10^-5 ^M dexamethasone, 1x ITS+1 premix (Sigma-Aldrich), and 10 ng/ml TGFβ1 (Humanzyme-Medicorp Inc.). For chondrogenesis in pellet culture, a total of 2.5 × 10^5 ^cells were spun in 1.5 ml sterile conical polypropylene microfuge tubes (Enzymax LLC, Kentucky, USA) at 1500 rpm for three minutes to form spherical cell pellets. The final volume of chondrogenic media was 250 μl per pellet. Medium change was performed three times a week. Hypoxia cultivated pellets experienced a short period (< 5 minutes) of exposure to normal oxygen tension during media changes. The pellets were cultured for three weeks under normal or low oxygen tension to allow appreciable matrix accumulation. Thereafter, the pellets were processed biochemically for glycosaminoglycan (GAG) and DNA contents, histologically, immunohistochemically and gene expression analysis via quantitative reverse transcription polymerase chain reaction (qRT-PCR) for cartilage specific matrix gene and proteins expression.

### Biochemical analysis

*In vitro *cultivated pellets were rinsed in PBS (Invitrogen) and were digested in proteinase K (1 mg/ml in 50 mM Tris with 1 mM EDTA, 1 mM iodoacetamide and 10 mg/ml pepstatin A; all from Sigma-Aldrich) for 16 hours at 56°C. The sulfated GAG content was measured by 1,9-dimethymethylene blue binding (Sigma-Aldrich) using chondroitin sulfate (Sigma-Aldrich) as standard. The DNA content was determined using the CyQuant cell proliferation assay kit (Invitrogen) with supplied bacteriophage λ DNA as standard. Statistical differences between test groups were evaluated by one-way analysis of variance (ANOVA) with Tukey's multiple comparison post-tests. Statistical analyses were performed using SPSS (version 18). A significant difference was considered when *P *< 0.05.

### Histology and immunohistochemistry

Tissues generated from the pellet cultures were fixed in 4% phosphate buffered formalin, processed into paraffin wax, sectioned at 5 μm and stained with 0.1% safranin O and counterstained with 1% fast green to reveal sulfated proteoglycan (GAG) matrix depositions. Other sections were probed with antibodies raised against collagen types I and II. Sections were treated with trypsin and then incubated with antibodies against collagen I (MAB3391, Millipore, Temecula, California, USA) or collagen II (II-II6B3 from Developmental Studies Hybridoma Bank at University of Iowa, USA). Immuno-localized antigens were visualized with goat anti-mouse IgG biotinylated secondary antibody (Dako Canada Inc, Mississauga, Ontario, Canada) and a streptavidin-horseradish peroxidase labeling kit with 3,3'-diaminobenzidine (Dako). Images were captured using an Omano OM159T biological trinocular microscope (Microscope Store, Virginia, USA) fitted with an Optixcam summit series 5MP digital camera and Optixcam software and assembled in Adobe Photoshop (Adobe Systems Inc. San Jose, USA).

### Gene expression analysis

Total RNA was extracted from pellets using Tri-Reagent (Sigma-Aldrich) after grinding with Molecular Grinding Resin (Geno Technology Inc. St Louis, USA) in combination with the use of an Aurum Total RNA Fatty and Fibrous Tissue Kit (Bio-Rad, Mississauga, Ontario, Canada) and after removal of contaminating genomic DNA from the pellets by DNase treatment. In order to mitigate changes in gene expression, the caps of pellet cultures were closed before removal from the low oxygen tension incubator and cell pellets were immediately transferred into Tri-Reagent. Total RNA (100 ng) in a 40 μl reaction was reverse transcribed to cDNA using GoScript reverse transcriptase (Fisher Scientific, Whitby, Ontario, Canada) primed in the presence of oligo dT primers (1 μg). Real-time quantitative polymerase chain reaction (qRT-PCR) was performed with a DNA Engine Opticon II Continuous Fluorescence Detection System (Bio-Rad) using hot start Taq and SYBR Green detection (Eurogentec North America Inc, San Diego, CA, USA). Primer sequences (Table 3) were taken from previously published work or were custom designed using the Primer Express software (Applied Biosystems, Foster City, California, USA) [23,34,35]. All primers were obtained from Invitrogen. Gene (mRNA) expression levels for each primer set were normalized to the expression level of human β-actin [9,34,36] by the 2-^Δct ^method [37]. Statistical differences between test groups were evaluated by one-way ANOVA with Tukey's multiple comparison post-tests. Statistical analyses were performed using SPSS (version 18). A significant difference was considered when *P *< 0.05.

**Table 3:** Primer sequences used in quantitative real-time PCR (all primers were purchased from Invitrogen, Mississauga, Ontario, Canada)

Primer			Reference
β-Actin	5'-AAGCCACCCCACTTCTCTCTAA-3'	(Forward)	[34]
	5'-AATGCTATCACCTCCCCTGTGT-3'	(Reverse)	
Aggrecan	5'-AGGGCGAGTGGAATGATGTT-3'	(Forward)	[34]
	5'-GGTGGCTGTGCCCTTTTTAC-3'	(Reverse)	
Collagen 1A2	5'-TTGCCCAAAGTTGTCCTCTTCT-3'	(Forward)	[34]
	5'-AGCTTCTGTGGAACCATGGAA-3'	(Reverse)	
Collagen 2A1	5'-CTGCAAAATAAAATCTCGGTGTTCT-3'	(Forward)	[34]
	5'-GGGCATTTGACTCACACCAGT-3'	(Reverse)	
Collagen 10A1	5'-CTGCAAAATAAAATCTCGGTGTTCT-3'	(Forward)	[23]
	5'-GGGCATTTGACTCACACCAGT-3'	(Reverse)	
COMP	5'-CCGACAGCAACGTGGTCTT-3'	(Forward)	[23]
	5'-CAGGTTGGCCCAGATGATG-3'	(Reverse)	
HIF-1α	5'-GTAGTTGTGGAAGTTTATGCTAATATTGTGT-3'	(Forward)	[35]
	5'-CTTGTTTACAGTCTGCTCAAAATATCTT-3'	(Reverse)	
HIF-2α	5'-GGTGGCAGAACTTGAAGGGTTA-3'	(Forward)	[30]
	5'-GGGCAACACACACAGGAAATC-3'	(Reverse)	
SOX9	5'-CTTTGGTTTGTGTTCGTGTTTTG-3'	(Forward)	[34]
	5'-AGAGAAAGAAAAAGGGAAAGGTAAGTTT-3'	(Reverse)	
TGFβ-RI	5'-GGCTTTTCTCCACATGCTTAGG-3'	(Forward)	[39]
	5'-GGCAACAGAGATCACCTGTAGACA-3'	(Reverse)	
TGFβ-RII	5'-CAGTGTGGCTGCAGTAGCATAGA-3'	(Forward)	[39]
	5'-CATGCCCTACGGTGCAAGT-3'	(Reverse)	

PCR, polymerase chain reaction.

### Ethical considerations

All studies were implemented with stem cells propagated from bone marrow aspirates taken from surgical discards of patients undergoing routine orthopedic procedures after approval and a waiver of informed consent of the local ethical committee of the University of Alberta (Edmonton, Canada).

## Results

### Effect of oxygen tension on the colony forming characteristics

**Figure 2:**
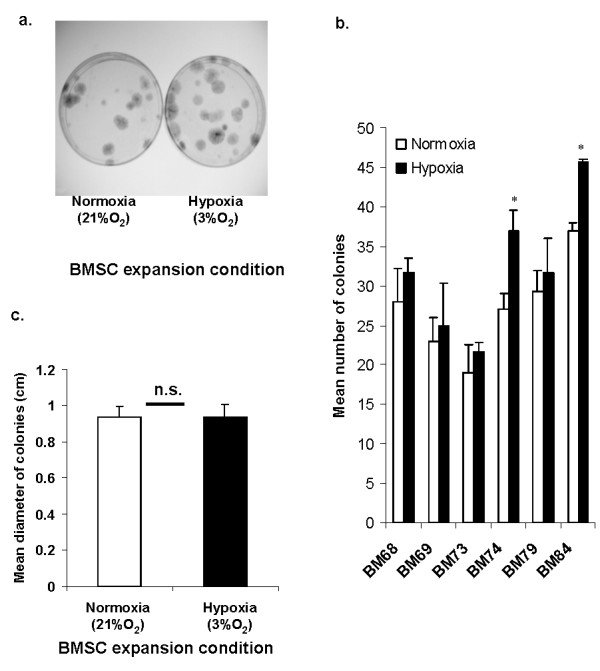
**(**A**) Representative photograph of colony forming unit fibroblastic (cfu-f) assay of passage 0 (P0) BMSC after 14 days of culture under normoxia or hypoxia**. Bone marrow mono-nucleated cells (MNCs) were seeded into 100 mm petri dish in triplicate (*n *= 3) at 250,000 MNCs per dish. Culture media was α-MEM comprising 10% FBS and 5 ng/ml FGF-2. Media were not changed within the first seven days. Thereafter, the media were changed twice a week. Developed cell colonies were visualized by crystal violet staining. (**B**) The number of cell colonies developed in the Cfu-f assay as described in A. The data represent mean ± standard error of colony counts derived from six donors (*n *= 3). (**C**) The mean diameter of cell colonies developed in the Cfu-f assay as described in A. The data represents mean ± standard error of colony counts derived from six donor specimens. Not significant (n.s.) = *P *> 0.05 or * *P *< 0.05 in normoxia versus hypoxia, Student's t test. BMSCs, bone marrow mesenchymal stromal cells; FBS, fetal bovine serum; FGF-2, fibroblast growth factor-2; MEM, modified Eagle's medium.

To investigate the colony forming characteristics of adherent BMSCs cultured under normoxia and hypoxia, the number and diameter of the cell colonies developed after 14 days of culture of bone marrow MNCs was evaluated (Figure 2). The number of adherent cell colonies developed was consistently higher under hypoxic conditions by 8% to 37% albeit with dependence on the donor (BM68 (13%), BM69 (9%), BM73 (14%), BM74 (37%), BM79 (8%) and BM84 (23%)) (Figure 2b). There was no significant difference between the measured diameters of the cell colonies under normoxia and hypoxia (Figure 2c).

### Effect of oxygen tension on the expression of cell surface markers

**Figure 3:**
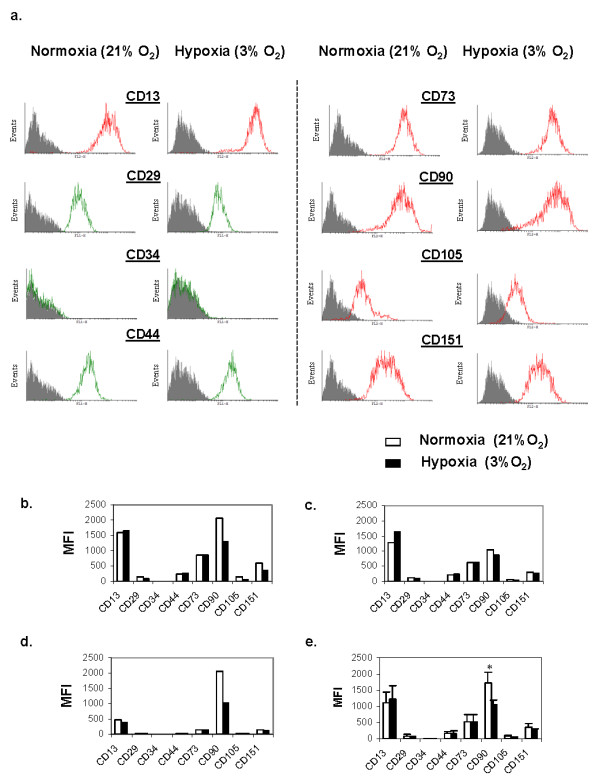
**Flow cytometry analysis of cell surface markers on passaged 2 (P2) BMSCs after culture in α-MEM comprising 10% FBS and 5 ng/ml FGF-2**. (**A**) Representative fluorescence intensity histograms are presented showing expression of cell surface markers of normoxia- and hypoxia-expanded BMSCs at P2 of a 43-year-old man (BM74). (**B-D**) The mean fluorescence intensity (MFI) of cell surface markers on BMSCs from: (B) a 34-year old woman (BM68), (C) a 43-year-old man (BM74), (D) a 62-year-old man (BM84). (**E**) Represents the mean fluorescence intensity ± standard error of three donors; BM68, BM74 and BM84. *P < 0.05 in normoxia versus hypoxia, Student's t test. BMSCs, bone marrow mesenchymal stromal cells; FBS, fetal bovine serum; FGF-2, fibroblast growth factor-2; MEM, modified Eagle's medium.

To investigate the immuno-phenotype of the adherent population of BMSCs isolated and expanded under normoxia and hypoxia, we analyzed the cell surface molecule expression from three different donors: BM68, BM73 and BM84. A panel of cell surface markers that are typically used to characterize mesenchymal stem cells was selected [25,38]. Both normoxia and hypoxia expanded BMSCs were positive for CD13, CD29, CD44, CD73, CD90, CD105 and CD151. Both normoxia and hypoxia expanded BMSCs were negative for CD34. The positive cell surface markers expressed on the adherent BMSCs displayed a single fluorescence peak binding profile by flow cytometry (Figure 3a). However, quantitative expression levels (MFI) of cell surface markers were dependent on donors and on the oxygen tension during cell expansion. For example, the expression level of CD90 was consistently and significantly lowered in hypoxia-expanded BMSCs compared to normoxia-expanded BMSCs (Figures 3b, c, d and 3e).

### Downstream effect of oxygen tension on extracellular matrix formation

**Figure 4:**
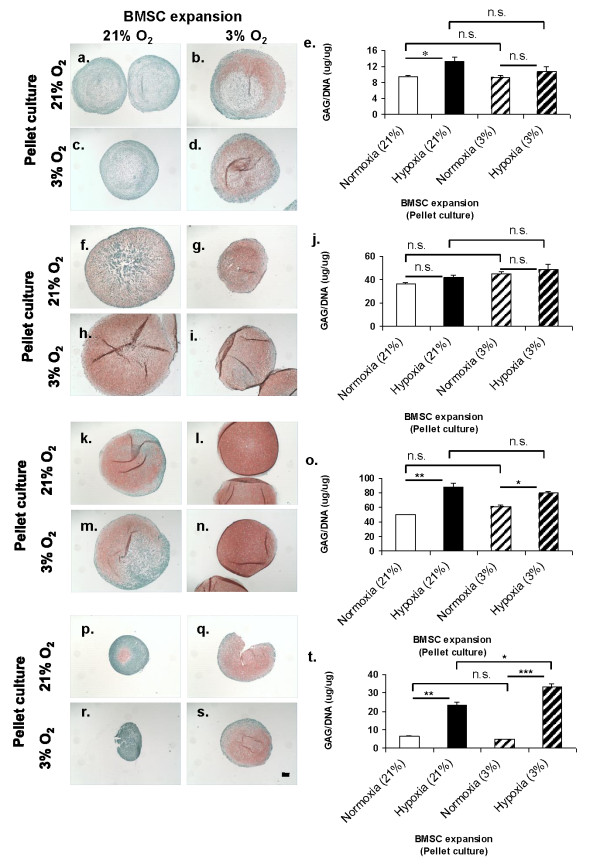
**Paraffin wax 5 μm-section pellets were stained with safranin-O/fast green stain**. All photomicrographs represent low (4x objectives) images. (**A-D**) 34-year-old woman (BM68), (**F-I**) 51-year-old man (BM73), (**K-N**) 43-year-old man (BM74) and (**P-S**) 62-year-old man (BM84). Scale bar is 100 μm. Biochemical assay of pellets for sulfated glycosaminoglycan (GAG) per DNA content. Pellets were cultured for three weeks in a defined serum-free chondrogenic medium under low (3%) or normal (21% O_2_) oxygen tension. Data are mean ± standard error of triplicates. (**E**) 34-year old woman (BM68), (**J**) 51-year-old man (BM73), (**O**) 43-year-old man (BM74) and (**T**) 62-year-old man (BM84). Data represent mean ± standard error of triplicate pellets; one-way ANOVA with Tukey's post hoc test: n.s. (not significant), * = *P *< 0.05, ** = *P *< 0.001 and *** = *P *< 0.0001. ANOVA, analysis of variance.

Safranin-O staining for sulfated GAG matrix deposition of pellets derived from four donors (BM68, BM73, BM74 and BM84) showed that the pellets derived from hypoxia-expanded BMSCs stained more strongly and more uniformly throughout the pellet than their counterparts from normoxia-expanded BMSCs, albeit with dependence on the donor (Figure 4). Furthermore, most pellets derived from normoxia-expanded BMSCs increased in safranin O staining under hypoxia, while pellets derived from hypoxia-expanded BMSCs remained intensely stained with safranin O. Quantitative GAG matrix normalized to DNA content of the pellets was determined (Figures 4e, j, o and 4t). The pellets formed from hypoxia-expanded BMSCs had a 1.4 to 3.5 fold increased GAG per DNA content than their counterparts from normoxia-mediated expansion, depending on the donor, when pellets were cultivated under normoxia while the pellets derived from hypoxia-expanded BMSCs had a 1.2 to 7.0 fold enhancement in GAG per DNA content than pellets from normoxia-expanded BMSCs when cultured under hypoxic conditions. These results correlated with the more uniform and intense safranin O staining observations in the pellets derived from hypoxia cultivated BMSCs. In the pooled data set, the mean GAG per DNA content of the pellets from hypoxia-expanded BMSCs was 1.7-fold (*P *< 0.05) higher than their normoxia counterparts under normoxic condition while under hypoxic condition. There was a linear-inverse relationship between the GAG per DNA contents of pellets and age of BMSC donors under a specific culture condition (data not shown).

### Downstream effect of oxygen tension on chondrogenic gene and protein expression, and TGFβ receptor expression

**Figure 5:**
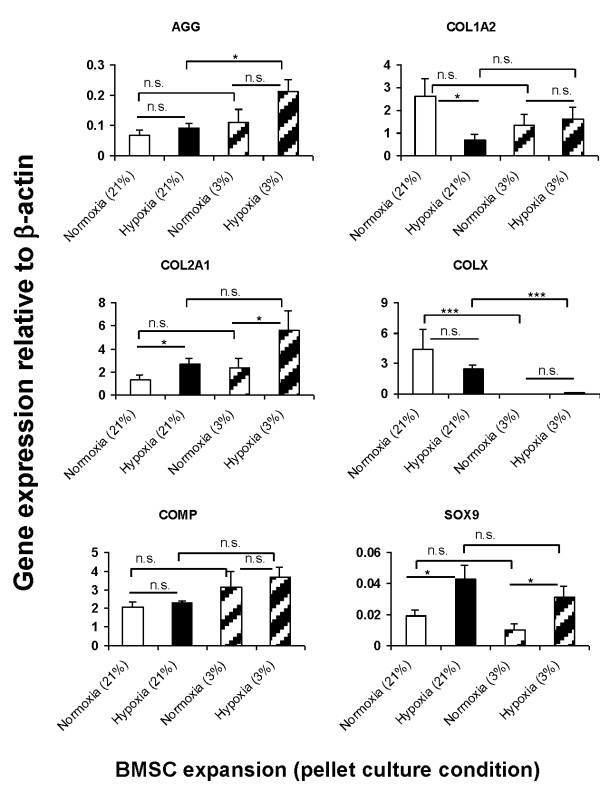
**(**A**) Real-time PCR analysis of cDNA derived from pellet cultures of normoxia- and hypoxia-expanded BMSCs**. Pellet cultures were performed under normoxic or hypoxic conditions for three weeks in the presence of defined serum-free chondrogenic factors as described Materials and Methods text. Real-time PCR analysis was *via *SYBR Green detection. Presented data represent the mean ± standard error of pellets pooled from four donors (that is, BM68, BM73, BM74 and BM84) in triplicate (*N *= 4, *n *= 12 per experimental group). Data represent mean ± standard error of triplicate pellets per donor; one-way ANOVA with Tukey's post hoc test: n.s. (not significant), * = *P *< 0.05, ** = *P *< 0.001 and *** = *P *< 0.0001. Gene expression is presented as relative mRNA level normalized to mRNA expression of human β-actin; *y*-axis. ANOVA, analysis of variance; BMSCs, bone marrow mesenchymal stromal cells; PCR, polymerase chain reaction.

**Figure 6:**
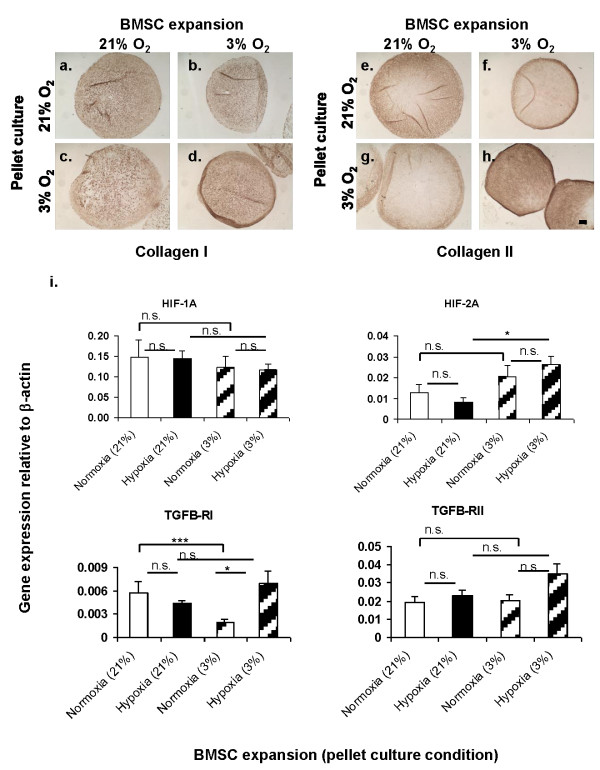
**(**A-H**) Immuno-labeling of 5 μm-sections of pellets with anti-collagen types I and II**. All photomicrographs represent low (4x objectives) images. Pellets were cultured for three weeks in chondrogenic medium under low (3%) or normal (21% O_2_) oxygen tension. Images were taken from pellets derived from BM73. Scale bar is 100 μm. (**I**) Real-time PCR analysis of cDNA derived from pellet cultures of normoxia- and hypoxia-expanded BMSCs. Pellet cultures were performed under normoxic or hypoxic conditions for three weeks in the presence of defined serum-free chondrogenic factors as described in the Materials and Methods text. Real-time PCR analysis was *via *SYBR Green detection. Presented data represent the mean ± standard error of pellets pooled from four donors (that is, BM68, BM73, BM74 and BM84) in triplicate (*N *= 4, *n *= 12 per experimental group). Data represent mean ± standard error of triplicate pellets; one-way ANOVA with Tukey's post hoc test: n.s. (not significant), * = *P *< 0.05 and *** = *P *< 0.0001. Gene expression is presented as relative mRNA level normalized to mRNA expression of human β-actin; *y*-axis. ANOVA, analysis of variance; BMSCs, bone marrow mesenchymal stromal cells; PCR, polymerase chain reaction.

Quantitative RT-PCR was performed for gene expression analysis on pellets derived from four donors (BM68, BM73, BM74 and BM84) after three weeks of chondrogenic culture. The mean relative gene expression of aggrecan (AGG), collagen I (*Col1a2*), collagen II (*Col2a1*), collagen X (*Col10a1*), cartilage oligomeric matrix protein (COMP), HIF-1α and HIF-2α, Sox9, TGFβ1-receptor I (TGFβ1-RI) and TGFβ1-receptor II (TGFβ1-RII) in the pooled pellets is presented in Figures 5 and 6. The mRNA expression levels of *Col2a1 *(2.44-fold) and Sox9 (2-fold) were significantly higher in pellets formulated from hypoxia-expanded BMSCs than in pellets derived from normoxia-expanded BMSCs after culture under normoxic conditions (Figure 5). The mean mRNA expression of AGG was not significantly higher in the pellets derived from hypoxia isolated and expanded BMSCs under normoxic conditions when compared to those derived from normoxia-expanded BMSCs (Figure 5). Under hypoxic culture condition, the mean mRNA expression level of AGG and *Col2a1 *were 2-fold higher while the mRNA expression of Sox9 was 3-fold higher in pellets derived from hypoxia-expanded BMSCs compared to their counterparts from normoxia-expanded BMSCs (Figure 5). The mean mRNA expression level of *Col1a2 *was significantly higher in pellets formulated from normoxia expanded BMSCs after culture under normoxic conditions while the expression of *Col10a1 *and COMP were not significantly different between the pellets from normoxia- and hypoxia-expanded BMSCs (Figure 5). In contrast, under hypoxic conditions of pellet culture the expression of *Col1a2*, regardless of the oxygen tension during BMSC expansion, was not significantly different between pellets (Figure 5). The expression of COMP increased under hypoxic pellet culture conditions relative to expression in pellets cultivated undr normoxia. However, the increment was not stastically significant. *Col10a1 *decreased significantly under hypoxic conditions of pellet culture. *Col10a1 *expression declined by 2225-fold in pellets formed from normoxia-expanded BMSCs and by 43-fold in pellets derived from hypoxia-expanded BMSCs when compared to their respective expression under normoxic conditions (Figure 5).

Immunohistochemical staining (brown coloration) of pellets from the four donors confirmed that the pellets formulated from normoxia- and hypoxia-expanded BMSCs expressed types I and II collagens. Representative photomicrographs of pellets (from donor BM74) labeled with antibodies to collagen I and II are shown in Figure 6a-6h. The pellets derived from hypoxia-expanded BMSCs were strongly and uniformly labelled with anti-collagen type II (Figures 6f (under normoxia) and 6h (under hypoxia)). In contrast, the pellets formulated from normoxia-expanded BMSCs labeled only strongly with anti-collagen type II at the pellet periphery and weakly in the central core (Figures 6e (under normoxia) and 6 g (under hypoxia)). Collagen type I was uniformly distributed in all pellets regardless of the oxygen tension during pellet culture or whether the pellets were formed from normoxia or hypoxia-expanded BMSCs (Figure 6a-6d). Primary antibody and non-specific IgG controls showed no false positive staining (data not included).

#### Differential expression of TGFβ receptor proteins

TGFβ-RI and TGFβ-RII have been implicated in the reduced chondrogenic potential of adipose derived mesenchymal stem cells under standard chondrogenic culture conditions using TGF-β1 or TGF-β3 [39]. We therefore investigated the expression of these receptor proteins in our pellets. The mRNA expression of TGFβ-RI and TGFβ-RII were statistically similar between the pellets derived from normoxia- and hypoxia-expanded BMSCs under normoxic conditions. However, under hypoxic conditions the expression of TGFβ-RI in pellets derived from hypoxia-expanded BMSCs were significantly up-regulated by 3.5-fold relative to its expression in pellets formed from normoxia-expanded BMSCs. Similarly, the mRNA expression of TGFβ-RII was increased under hypoxic condition in pellets derived from hypoxia-expanded BMSCs; however, the increment was not statistically significant relative to its expression in pellets formed from normoxia-expanded BMSCs (Figure 6i).

The cellular response to low oxygen tension in many mammalian cells is regulated by the transcriptional activity of HIF-1, a heterodimer of HIF-1α and HIF-1β [40]. HIF-2, a heterodimer of HIF-2α and HIF-1β, has also been identified as a regulator of the response of mammalian cells to low oxygen tension [41,42]. In addition, both HIF-1 and HIF-2 have been implicated in hypoxia-mediated enhancement of chondrogenesis of mesenchymal cells [29,30,41-43]. Thus, we investigated the gene expression of HIF-1α and HIF-2α in our study pellets after cultivation under normoxic and hypoxic conditions. The mRNA expression level of HIF-1α was similar in pellets regardless of whether the pellets were formed from normoxia- or hypoxia-expanded BMSC or cultivation of pellets under normoxic or hypoxic conditions (Figure 6i). The expression of HIF-2α was the same in pellets derived from normoxia- and hypoxia-expanded BMSCs under normal oxygen tension. However, under low oxygen tension the expression of HIF-2α increased regardless of whether the pellets were derived from normoxia- or hypoxia-expanded BMSCs. The expression of HIF-2α increased significantly by 3.2-fold in pellets derived from hypoxia-expanded BMSCs when compared to its expression in pellets under normoxic conditions. In contrast, the increment determined (1.6-fold) in pellets derived from normoxia-expanded BMSCs under hypoxic conditions relative to its expression in the same pellets under normoxic conditions was not statistically significant (Figure 6i).

## Discussion

In this study we have compared the chondrogenic potential of human BMSCs obtained under low (3%) and normal (21%) oxygen tension. The BMSCs were isolated *via *plastic adherence and cell culture mediated propagation. We have used flow cytometry to characterize the cell surface protein expression of the BMSCs and used an *in vitro *pellet model of chondrogenesis with TGF-β1 to investigate their capacity to undergo chondrogenic differentiation.

Our data showed that the BMSCs obtained after plating of bone marrow nucleated cells and subsequent cell culture propagation under low oxygen tension expressed a panel of conventionally used mesenchymal stem cell surface markers but with a consistently reduced concentration (that is, reduced mean fluorescence intensity) of CD90. The BMSCs obtained *via *propagation under low oxygen tension underwent a more robust chondrogenesis than their counterparts under normal oxygen tension albeit with dependence on the donor. The reduced CD90 expression in the BMSCs harvested under hypoxia may be associated with improved chondrogenesis. A link between CD90 expression and chondrogenic potential of BMSCs has not been reported in the literature. However, the lack of CD90 expression in *in vitro *expanded human articular chondrocytes has been observed with chondrocytes with higher chondrogenic potential [44]. Thus, there is reason to speculate on a potential link between its expression and the chondrogenic capacity of BMSCs. Furthermore, *in vitro *expanded human articular chondrocytes have also been reported to display plasticity features that are similar to those of mesenchymal stem cells [45]. Regardless of the oxygen tension during pellet culture, the BMSCs isolated and propagated under hypoxic conditions displayed a superior chondrogenic capacity than their counterparts from normal oxygen tension. The superior chondrogenic capacity was characterized by higher GAG per DNA content, enhanced transcript expression of a panel of chondrogenic genes (aggrecan, *Col2a1 *and Sox9) as well as the intense and uniform distribution of collagen II and safranin O staining for sulfated proteoglycans. Our data mirrors the findings of Tew *et al. *[46], that demonstrated that pellets derived from de-differentiated and Sox9 transduced articular chondrocytes displayed a phenotype that was consistent with a more robust chondrogenic response (increased safranin O staining and *Col2a1 *expression) compared to control pellets formed from non-transduced and de-differentiated articular chondrocytes. Furthermore, our data of increased mRNA expression of Sox9 and aggrecan supports previously reported evidence that transcriptional activity of Sox9 enhances the gene promoter activities of aggrecan in chondrocytes [46-49]. Transcriptional factors Sox9, L-Sox5 and Sox6 have been reported to be essential for regulating the expression of *Col2a1 *as well as other genes involved in chondrogenesis [48,50,51].

The chondrogenic capacity of all pellets in our study improved further under low oxygen tension. The improvement was characterized by a marginal (that is, non-statistically significant) increase in transcript expression of aggrecan, *Col2a1 *and COMP. Furthermore, and more significantly, the improvement in chondrogenic potential under hypoxia was accompanied by a concomitant suppression in *Col10a1 *expression, a marker of hypertrophic chondrogenesis and terminally differentiated chondrocytes. This finding is in accordance with the observation of Hirao *et al. *that hypoxia (that is, 5% O_2_) supports commitment of C3HT10T1/2 (a pluripotent mesenchymal cell line) to chondrogenic differentiation rather than osteogenesis through mechanisms involving a concomitant down-regulation of *Col10a1 *and Runx2 activity *via *Smad suppression and histone deactylase 4 activation (HDAC4) [52].

Our results indicate that the response of BMSCs to hypoxic conditions involves up-regulation of the transcriptional expression of HIF-2α rather than HIF-1α, which remained unperturbed. This finding was surprising since hypoxia has been reported to induce chondrocyte-specific gene expression (aggrecan, *Col2a *and Sox9) in mesenchymal cells (mouse ST2 stromal cells and C3HT10T1/2) through transcriptional activity of HIF-1α [53]. Furthermore, HIF-1α, has been implicated in hypoxia-mediated inhibition of senescence and maintenance of human mesenchymal stem cell properties [43]. Our finding, therefore suggests that the response of the BMSCs to hypoxia was mediated by the transcriptional activity of HIF-2α. The involvement of HIF-2α here is in accordance with a report that hypoxia (that is, 5% O_2_) enhances the expression of several chondrogenic genes including *Col2a1*, aggrecan and Sox9 during chondrogenesis of adipose derived mesenchymal stem cells [30]. However, in contrast to the hypoxia-mediated down-regulation of *Col10a1 *noted in our present study, hypoxia (5% O_2_) enhanced the expression of *Col10a1 *during the chondrogenic differentiation of adipose derived mesenchymal stem cells [30]. A plausible explanation for the differential outcome may depend on the difference in oxygen tension or cell type. Nonetheless, our data opens the perspective of a possible mechanistic link between the transcriptional activity of HIF-2α and collagen X expression.

### Differential expression of TGFβ-receptors

TGFβ-RI and TGFβ-RII have been implicated in the reduced chondrogenic potential of adipose derived mesenchymal stem cells relative to the chondrogenic potential of BMSCs. Thus, we investigated the expression of these receptor proteins in our BMSCs pellets. Our data showed that the expression of these receptors was generally higher in pellets derived from hypoxia-expanded BMSCs after culture in hypoxic conditions. However, it was surprising that the expression of TGFβ-RI was statistically higher in pellets formulated from hypoxia-expanded BMSCs during pellet culture under hypoxic conditions.

## Conclusions

We have shown that the isolation and expansion of BMSCs under hypoxic conditions of 3% oxygen tension increases the propensity of the BMSCs to undergo a more robust chondrogenesis under normoxic and hypoxic culture conditions relative to BMSCs isolated and propagated under normoxic conditions. Our results also show that the response of these cells to low oxygen tension is mediated by HIF-2α. Taken together, our finding highlights the need to isolate and propagate BMSCs under hypoxic conditions for improved *in vitro *chondrogenesis and for *in vivo *cartilage repair and/or regeneration. The outcome of this study suggests that oxygen tension is an important factor in the determination of the chondrogenic differentiation fate of adherent populations of BMSCs.

## Abbreviations

AGG: aggrecan; BMSCs: bone marrow mesenchymal stromal cells; β-actin: beta actin; cDNA: complementary deoxyribonucleic acid; COL1A2: type I collagen α2 chain; COL2A1: type II collagen α1 chain; COMP: cartilage oligomeric matrix protein; ECM: extracellular matrix; FGF-2: basic fibroblast growth factor; GAG: glycosaminoglycan; HIF-1α: hypoxia inducible factor-1 alpha; HIF-2α: hypoxia inducible factor-2 alpha; MNC: mononuclear cells; mRNA: messenger ribonucleic acid; MSC: mesenchymal stem cells; OA: osteoarthritis; qRT-PCR: quantitative real-time polymerase chain reaction; SOX9: Sry-related HMG box-9; TGF-β1 or β3: transforming growth factor -β1 or β3; TGFβ-RI: transforming growth factor β receptor I; TGFβ-RII: transforming growth factor β receptor II.

## Competing interests

The authors declare that they have no competing interests.

## Authors' contributions

ABA conceived and designed the study, performed experiments, data acquisition and analysis, manuscript writing and supervision of the entire study. AMS performed experiments and data acquisition. NMJ was responsible for bone marrow procurement, data analysis and manuscript writing. All authors read and approved the final manuscript.

## Acknowledgements

We would like to thank Dr Thomas Churchill and Jacek Studzinski (Department of Surgery, University of Alberta, Canada) for histological assistance. Financial support was provided in part by: Edmonton Orthopaedic Research Committee, University of Alberta Hospital Foundation to AA, Edmonton Civic Employee's Charitable Fund to AA and new investigator startup fund by Department of Surgery, University of Alberta to AA.
